# Supplementary material for: Clinical Significance of the Immunohistochemical Expression of Histone Deacetylases (HDACs)-2, -4, and -5 in Ovarian Adenocarcinomas
Source: Biomedicines. 2024 Apr 24;12(5):947. doi: 10.3390/biomedicines12050947 (PMC11118868; doi:10.3390/biomedicines12050947)

Figure S1. Different staining intensities in the expression of HDAC-2, HDAC-4 and HDAC-5 in ovarian carcinomas.

A. Mild HDAC-2 expression (x400)
B. Moderate HDAC-2 expression (x400)
C. Intense HDAC-2 expression (x400)
D. Mild HDAC-4 expression (x400)
E. Moderate HDAC-2 expression (x400)
F. Mild HDAC-5 expression (x400)
G. Moderate HDAC-5 expression (x400)
H. Intense HDAC-5 expression (x400)

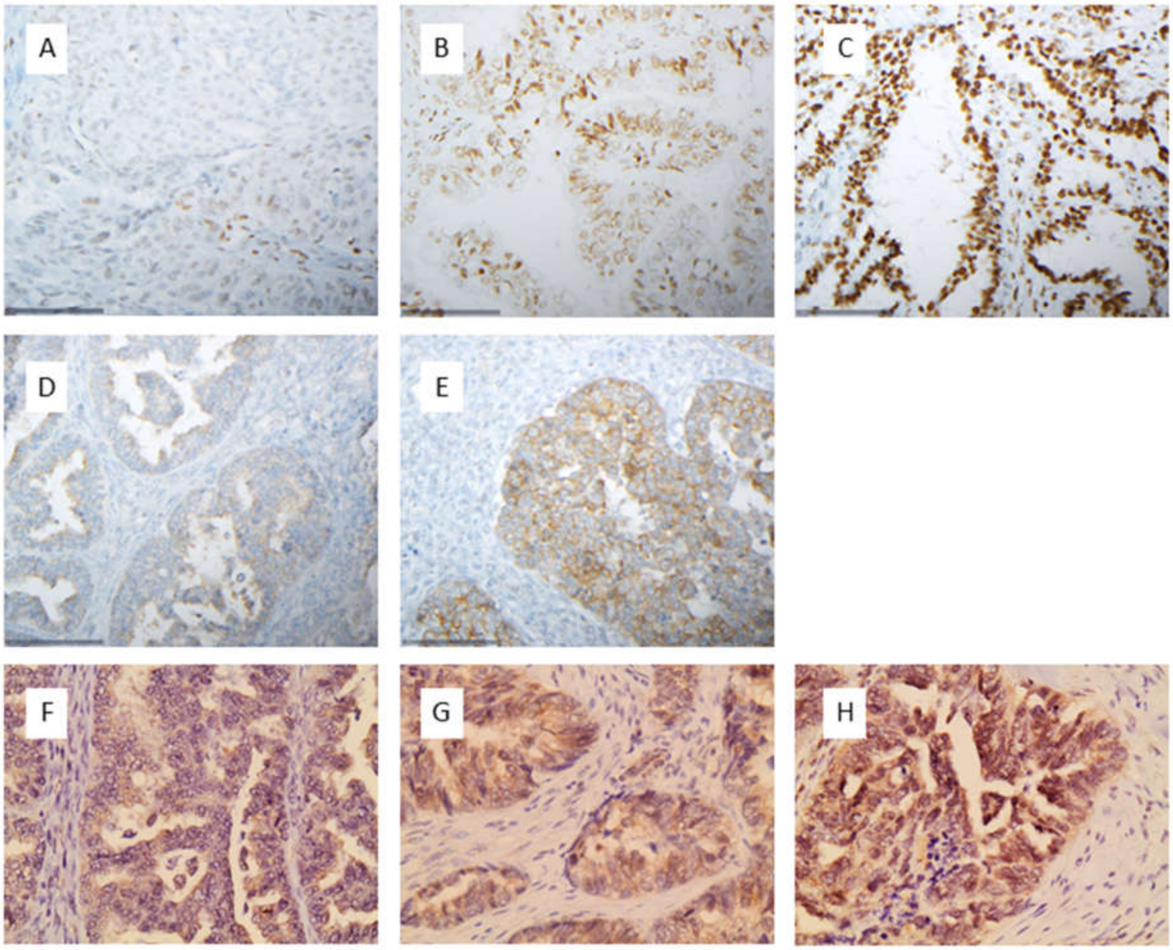

Figure S2. Graphical representation of the association between HDAC-4 and cytoplasmic HDAC-5 expression (first panel) as well as between HDAC-4 and nuclear HDAC-5 expression (second panel).

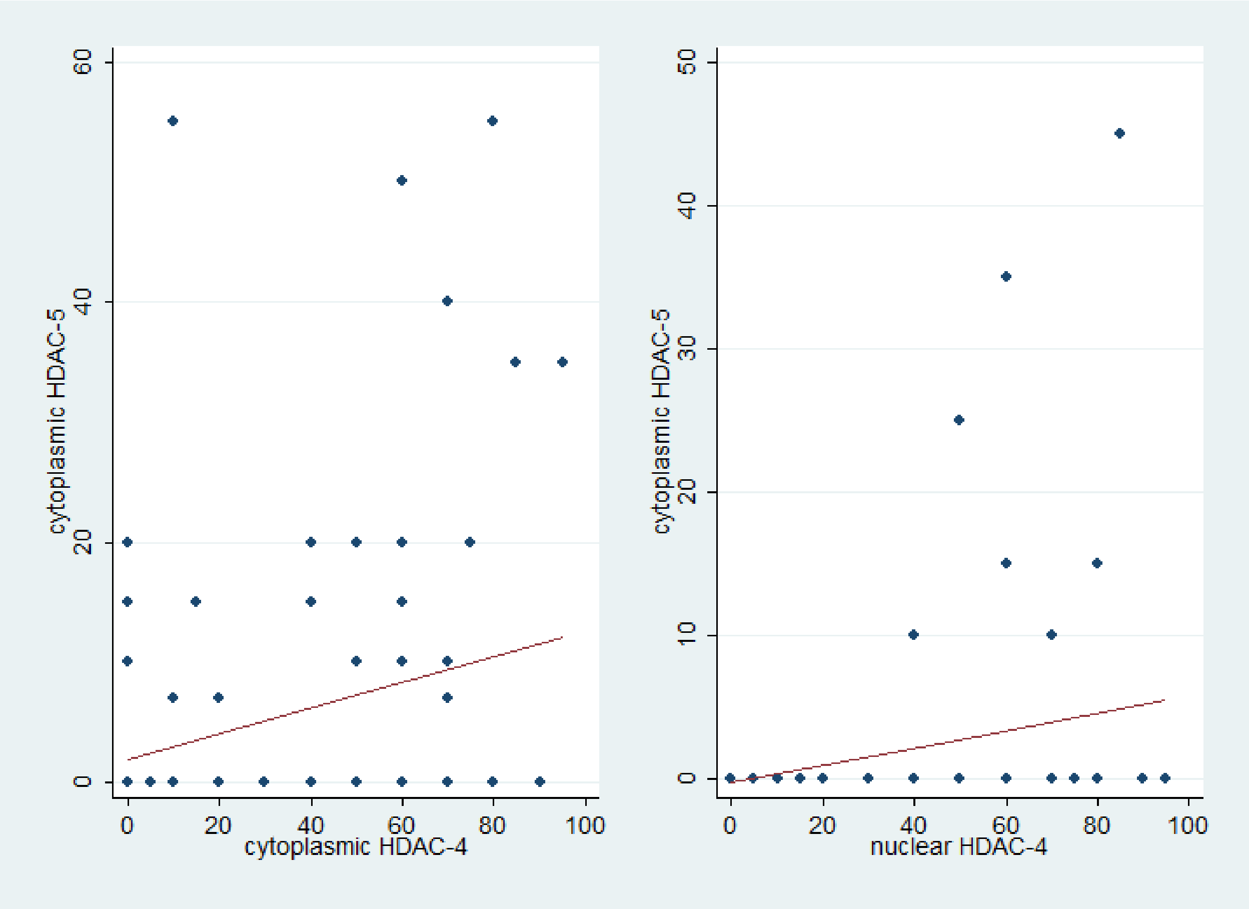

Supplement: Supplementary file 1 [file biomedicines-12-00947-s001.zip › biomedicines-2955760-supplementary.pdf]
